# Urbanization Affects Soil Microbiome Profile Distribution in the Russian Arctic Region

**DOI:** 10.3390/ijerph182111665

**Published:** 2021-11-06

**Authors:** Maria V. Korneykova, Viacheslav I. Vasenev, Dmitry A. Nikitin, Anastasia S. Soshina, Andrey V. Dolgikh, Yulia L. Sotnikova

**Affiliations:** 1 Agrarian and Technological Institute, Peoples’ Friendship University of Russia (RUDN University), 117198 Moscow, Russia; slava.vasenev@wur.nl (V.I.V.); sotnikova-yul@rudn.ru (Y.L.S.); 2 Institute of North Industrial Ecology Problems, Subdivision of the Federal Research Centre, Kola Science Centre of Russian Academy of Sciences, 184209 Apatity, Russia; anastasiya.soshina97@yandex.ru; 3 Soil Geography and Landscape Group, Wageningen University, 6707 Wageningen, The Netherlands; 4 V.V. Dokuchaev Soil Science Institute, Russian Academy of Sciences, 119017 Moscow, Russia; dimnik90@mail.ru; 5 Institute of Geography, Russian Academy of Sciences, 119017 Moscow, Russia; dolgikh@igras.ru

**Keywords:** prokaryotes, fungi, biomass, quantitative PCR, soil profile, urban ecosystems, Arctic

## Abstract

Urbanization in the Arctic results in considerable and still poorly known environmental consequences. The effect of urbanization on soil microbiome—an ecosystem component highly sensitive to anthropogenic disturbance—remains overlooked for the Arctic region. The research compared chemical and microbial properties of the natural Podzol soils and urban soils of Murmansk—the largest Arctic city. Particular attention was given to the profile distribution, which is almost completely ignored by most microbial studies. Soil microbiome was investigated by the quantitative indicators based on fluorescence microscopy (microbial biomass) and PCR real-time methods (amount of rRNA genes copies of archaea, bacteria, and fungi). The principal changes in urban soils’ properties compared to the natural references included a shift in pH and an increase in C and nutrients’ contents, especially remarkable for the subsoil. The numbers of rRNA genes copies of archaea, bacteria, and fungi in urban topsoils (10^6^–10^10^, 10^9^–10^10^, and 10^7^–10^9^, respectively) were lower than in Podzol; however, the opposite pattern was shown for the subsoil. Similarly, the total microbial biomass in urban topsoils (0.55–0.75 mg g^−1^) was lower compared to the 1.02 mg g^−1^ in Podzols, while urban subsoil microbial biomass was 2–2.5 times higher than in the natural conditions. Both for urban and natural soils and throughout the profiles, fungi were dominated by mycelium forms; however, the ratios of mycelium–spores were lower, and the amount of thin mycelium was higher in urban soils than in natural Podzols. Urbanization in the Arctic altered soil morphological and chemical properties and created a new niche for microbial development in urban subsoils; its contribution to biodiversity and nutrient cycling promises to become increasingly important under projected climate change.

## 1. Introduction

Arctic and Subarctic regions attract the increasing attention of researchers and policy-makers due to the high vulnerability of ecosystems to global changes [1,2]. The negative effects of climate change on Arctic and Subarctic ecosystems are regularly presented by global reports [3] and regional studies [4,5,6]. The environmental consequences of urbanization in the Arctic are overlooked so far; however, changes in Arctic vegetation and soils driven by urbanization can be irreversible and dramatic [7,8]. Industrial and mining activities result in severe environmental pollution, which affects the vegetation state and diversity [9] and soil quality [10,11]. At the same time, the urbanization effect on soils is not limited to pollution. Constructing urban soils (e.g., to support green infrastructures) coincides with additional input of organic materials: peat, compost, and topsoil relocated from arable lands [12,13]. Soil management improves soil structure, changes pH, and increases nutrient content [14]. Indirectly, soils are also affected by urban heat islands [15,16], which create a more favorable temperature and moisture regime in comparison to the natural soils of the Arctic region.

Soil microorganisms are highly sensitive to environmental conditions [17]; therefore, considerable changes in soil microbiome can be expected as a result of urbanization. Soil microorganisms are responsible for many important functions and ecosystem services, including mineralization of organic matter [18,19,20] and supporting nutrient cycling [21,22] and biodiversity [23,24]. The effect of urbanization on these functions is still poorly understood for the Arctic regions. So far, the studies of the urban soil microbiome in the Arctic are rare and mainly limited to the identification of the pathogenic species to assess sanitary and epidemiological risks [25,26]. Although an increase of species pathogenic for humans in soils of Arctic cities is an important problem [27], a broader view on microbial diversity and activity is needed to understand the transformations in soil microbes induced by urbanization. As a rule, soil microbial studies in the Arctic are focused on the top 5–10 cm, where the highest biomass and diversity is expected [28]. This assumption is robust for the natural soils dominated by Podzols with a shallow organic horizon [29]. However, in the urban soils where the deeper layers can be strongly anthropogenically affected or even artificially constructed, the profile distribution of microbial properties is highly relevant [30].

Murmansk, located in Russia on the Kola Peninsula, is considered the biggest Arctic city in the world. A few studies on the soils of Murmansk were focused on the chemical properties rather than on soil microbiota [31]. Soil microbiomes of this polar city were examined only by ecotoxicological studies and sanitary investigations [32], while the aspects of microbial ecology remained overlooked. The existing results were obtained by classical methods of microbiological inoculation, concerned only cultivated bacteria, and described only the topsoil layer, whereas changes in microbial properties down the soil profile were never investigated.

Our research aimed to investigate microbial properties, including the microbial biomass structure, content of ribosomal genes of prokaryotes and fungi, and their distribution down the urban and natural soil profiles in the Murmansk city.

## 2. Materials and Methods

### 2.1. Research Area

Murmansk (68°58′ N 33°05′ E) is located in the North of the Kola Peninsula on the east coast of the Kola Bay. The area belongs to the Atlantic-Arctic zone of temperate climate due to the influence of the warm current of the Gulf Stream. The average temperature of the coldest months is about −11 °C, which is considerably warmer than in general for the Kola Peninsula, and the average temperature of the summer months is +13 °C. A major part (up to 68%) of the annual 500 mm precipitation occurs as snow during the winter period [33]. The updated Koppen–Geiger climate class [34] is Subarctic, Dfc (cold, no dry season, cold summer). The lithology of the area is formed by massive crystallic granites and gneisses of the Baltic shield covered by moraine deposits, which results in a hilly terraced relief. The area belongs to the forest-tundra zone, which stretches in a narrow strip about 50 km wide parallel to the coast of the Barents Sea. Podzols are the dominating zonal soil type [35].

Founded at the beginning of the 20th century as a harbor on the Barents Sea, today, with a population above 280,000, Murmansk is recognized as the largest world city beyond the Arctic circle. Land cover analysis based on remote sensing showed that less than 20% of the city territory was sealed (covered by impervious surfaces, including buildings and roads), whereas trees and shrubs covered the first half of the area [36]. The central part of the city is dominated by low-rise buildings, including houses constructed from wood in the first half of the 20th century. The suburbs are dominated by 5–10-floor apartment buildings constructed during the last 30 years.

### 2.2. Soil Survey

**Figure 1:**
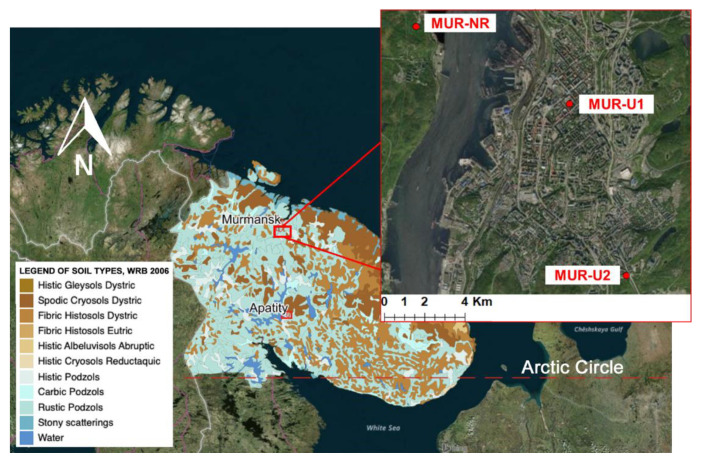
Research area and sampling scheme.

A soil survey was carried out in 2020 and included two locations in the city of Murmansk (MUR-U1 in the center and MUR-U2 in the suburb) and the third location in the natural forest-tundra area (MUR-NR), considered as a natural reference (Figure 1). At each location, a soil pit was excavated for soil description, morphological analysis, and classification according to World Reference Base [37]. Two additional points were sampled by augering, giving in total three sampling points per location. From each sampling point, the mixed samples were taken from each soil genetic horizon.

For the chemical analysis, the samples were taken from different soil horizons, transported to the lab, air-dried (22 °C), and sieved (mesh 2 mm). For the microbiological analysis, the samples were collected from the same horizons according to the standard sampling procedure with possible measures to prevent contamination [38].

### 2.3. Soil Physical and Chemical Properties

For each sampling points, three mixed samples (centre and corners) were taken for further analysis, giving in total nine mixed samples from each location for each horizon. Bulk density samples were collected by steel rings with the standard volume (100 cm^3^) and oven-dried at 105 °C. Bulk density (g cm^−3^) was calculated as the ratio of the oven-dried mass and the volume of the ring. Soil texture classes were indicated in the field based on the finger test and further adjusted in the lab following the standard procedure [39,40]. The pH value (soil–water = 1:5) was measured by electrometric technique (pH-meter Starter). Soil organic carbon content (SOC) was measured by dichromate oxidation (K_2_Cr_2_O_7_–H_2_SO_4_ = 1:1, 150 °C) with photometric determination of Cr^3+^ [41]. Total carbon and nitrogen contents were measured by combustion in a CN analyzer (Vario TOC Elementar). Phosphorus content was measured by the spectrophotometric technique (HACH DR-3900), and potassium content was measured on a flame photometer. Content of heavy metals (Ni, Cu, Pb, Zn, Cd, and Co) extracted by 5.0 M HNO_3_ were determined by inductively coupled plasma atomic emission spectrometry [42].

### 2.4. Soil Microbiological Properties

Prokaryotic biomass was estimated by using acridine orange, fluorescent dye (microscope “Biomed 5 PR LUM” (Russia)) at a magnification of 1000× with oil immersion [43]. Desorption of cells from the soil was carried out using a UZDN-1 ultrasonic unit (2 min, current 0.40 A, frequency 22 kHz). The calculation of the number of prokaryotic cells per 1 g of the substrate was carried out according to the formula:

N = S_1_ × a × n/V × S_2_ × C, (1)

where N is the number of cells per 1 g of the substrate; S_1_ is the area of the preparation (μm^2^); a is the number of cells in one field of view (averaging is performed over all preparations); n is the indicator of the dilution of the bacterial mixture (mL); V is the volume of the drop applied to the glass (mL); S_2_ is the area of the field of view of the microscope (μm^2^); C is the weight of the substrate, g.

The length of the actinomycete mycelium in 1 g of the sample (NMA) was determined by the formula:

NMA = S_1_ × a × n/v × S_2_ × c × 10^6^, (2)

where: S_1_ is the area of the preparation (μm^2^); a is the average length of fragments of actinomycete mycelium in the field of view (μm); n is the dilution index of the suspension (mL); v is the volume of the drop applied to the glass (mL); S_2_ is the area of the microscope field of view (μm^2^); c is the sample weight (g).

Fungal biomass was determined by fluorescence microscopy using the fluorescent dye calcofluor white (KB) [43]. The spores and the length of the mycelium were counted on a Biomed 5 PR LUM (Russia) fluorescent microscope at a magnification of 400×. Desorption of cells from the soil was carried out using a vortex “MSV-3500” (Latvia) at a speed of 3500 rpm for 10 min.

The calculation of the number of fungal cells per 1 g of the substrate was carried out according to the formula:

M = ((4 × a × n)/p) × 10^10^, (3)

where M is the number of cells in 1 g of soil; a is the average number of cells in the field of view; p is the area of the field of view (μm^2^); n is the dilution index.

The length of the mushroom mycelium in 1 g of the sample (NMA) was determined by the formula:

NMA = S_1_ × a × n/v × S_2_ × c × 10^6^, (4)

where S_1_ is the area of the preparation (μm^2^); a is the average length of mycelium fragments in the field of view (μm); n is the dilution index of the suspension (mL); v is the volume of the drop applied to the glass (mL); v is the volume of the drop applied to the glass (mL); S_2_ is the area of the microscope field of view (μm^2^); c is the sample portion (d).

Fungal biomass (mg/g soil) was calculated assuming that the spore density is 0.837 g/cm^3^, and the mycelium density is 0.628 g/cm^3^ [44]. The content of fungal biomass per gram of dry soil was calculated considering its moisture content.

Each soil sample was examined by fluorescence microscopy in six physical replicates. In each physical replication of the soil sample, 90 fields of view were examined under a microscope.

Total DNA was extracted from weighed soil samples (0.2 g) using a PowerSoil DNA Isolation Kit (MO BIO Laboratories, Carlsbad, CA, USA) according to the manufacturer’s protocol. Before DNA extraction, the samples were stored at −70 °C. Primary processing of soil samples was carried out using a Precellys 24 homogenizer (Bertin Technologies, Bertonneux, France). The quantitative assessment of the content of ribosomal genes of bacteria, archaea, and fungi was carried out by the method of polymerase chain reaction (PCR) in real time. To count archaea and bacteria, primers were used for the 16S rRNA gene, and for fungi, for the ITS region were quantified using primer sets described in Table 1. The reaction was carried out in a Real-Time CFX96 Touch amplifier (Bio-Rad Laboratories, Hercules, CA, USA). The reaction mixture was prepared from SuperMix Eva Green (Bio-Rad, Hercules, CA, USA). Solutions of cloned fragments of *Escherichia coli* K12, *Halobacterium salinarum* FG-07, and *Saccharomyces cervisiae* Meyen 1B-D1606 were used as quantitative standards for the concentrations of bacterial 16S rRNA genes, archaeal 16S rRNA genes, and fungal ITS genes, respectively. For each sample, the reaction was carried out in three replicates. Gene concentration was calculated using CFX Manager software. The number of genes in the DNA preparations was recalculated per gram of soil, considering the dilutions and the weight of the sample.

Statistical analyses were carried out in the R 4.0.3 software package. To determine the reliability of differences in experimental data, all obtained samples were tested for normal distribution using the Shapiro–Wilk normality test. If the data were normally distributed, then the variances were compared by Fisher’s test (F-test). If the variances were not equal, then the significance of the differences was determined by the Student’s criterion. If the variances are equal, then the significance of the differences was determined by Welch’s criterion. If the data were not normally distributed, then, the samples as a whole were compared using the Wilcoxon–Mann–Whitney test. The reliability of the influence of chemical parameters on biological ones was determined using one-way analysis of variance (ANOVA) and correlation analysis using Pearson’s test. The significance level was 0.05.

## 3. Results

### 3.1. Soil Morphological and Chemical Properties

**Figure 2:**
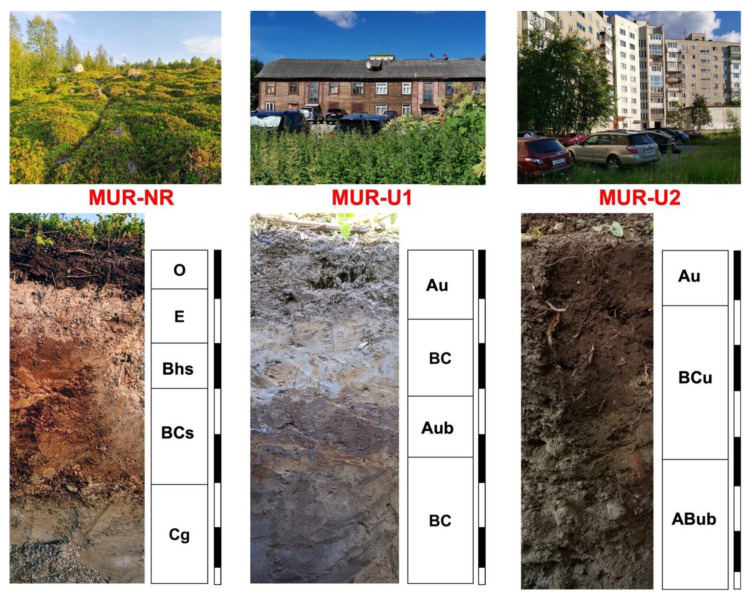
Sampling locations and soils profiles (black and white lines correspond to 10 cm depths).

The natural soil profile (MUR-NR) was classified as Folic Leptic Albic Podzol (Arenic), based on the detected spodic (Bs) horizon and E horizon with albic material formed as a result of the vertical migration of sesquioxides and organic matter. Slow decomposition of the plant residuals in the Subarctic climate resulted in the formation of a 7 cm folic (O) horizon. A high fraction of rock fragments in the moraine parent material limited vertical water fluxes and resulted in a slight stagnic feature in the BCs and Cg horizons. The morphological properties of the urban soils, classified as Urbic Technosols (Arenic) based on the number of artifacts (e.g., bricks, coals, gravel, and wastes from building construction), were considerably different from the natural soil. Alteration of the natural forest-tundra vegetation with moss in the surface layer into green lawns and ruderal grasses with ornamental trees and shrubs changed the amount and structure of carbon input, whereas higher surface temperature increased the decomposition rate. As a result, a humus-accumulative horizon Au was detected in both urban soils’ profiles. In the relatively young MUR-U2 site, the Au was underlain by the technogenic subsoil transporting BCu horizon (a sandy layer transported for the building construction) and the buried urbic insitu-formed ABub horizon. The profile MUR-U1 located in the city center was likely exposed to the two consequent stages of urbanization, reflected in the buried humus-accumulative Aub horizon. Apparently, the initial urban soil profile was covered by the excavated BC horizon for leveling or other land engineering purposes. The top Au horizon was formed on top of it as a result of humification and mineralization of the ruderal grasses, which dominate the courtyard surface today (Figure 2). Thus, the three investigated profiles illustrated the effect of urbanization on soil formation and functioning in the Subarctic conditions, which was further observed in the profile distribution of soil chemical and microbiological properties.

### 3.2. Chemical Properties

**Figure 3:**
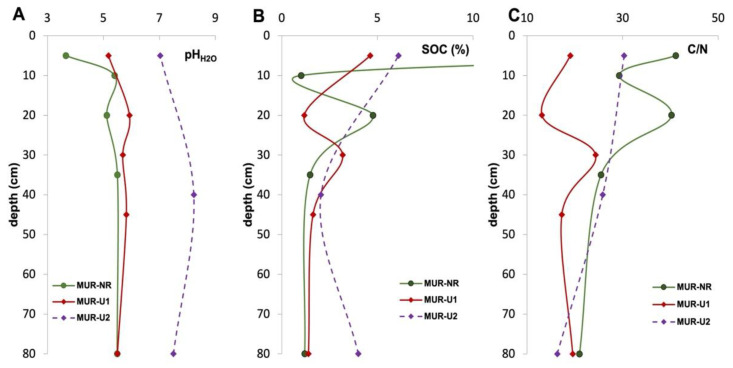
Changes in pH_H2O_ (**A**), SOC (**B**), and C/N (**C**) down the profile of urban and natural soils.

Folic Leptic Albic Podzol (Arenic) under forest-tundra had highly acidic pH_H2O_ in the O horizon and slightly acidic pH_H2O_ in the subsoil horizons with the maximum value in BCs. In MUR-U1 Urbic Technosol, pH_H2O_ was on average one unit higher than in the natural soil, with the highest values in the middle part of the profile and the lowest values in the topsoil and parent material. In contrast to the first two profiles, pH_H2O_ in MUR-U2 Urbic Technosol ranged from neutral to slightly alkaline, with the average values three units higher than in the natural conditions (Figure 3A). Profile distribution of SOC in MUR-NR had two maximums—in O and Bhs horizons, which is typical for Podzols [28,35]. Similarly, SOC distribution in the MUR-U1 profile had two maximums; however, in this case, they corresponded to the surface and buried Aur horizons. In the MUR-U2 profile, SOC stocks in the top Au and buried urbic ABub horizons were comparably high, whereas a considerably smaller amount of SOC was stored in the technogenic BCu horizon (*p* = 0.01399). In general, urban soils contained less SOC than the natural Podzol in the top 5–10 cm; however, the subsoil SOC contents were higher in the urban soils (Figure 3B). The profile distribution of the C/N ratio in Podzols was similar to the SOC distribution, with the highest values obtained for the O and Bhs horizons. The maximum C/N in MUR-U1 was obtained for the buried Aub horizon. A higher value compared to the surface Au horizon can be explained by a higher N input from current soil management compared to the previous urbanization stage. A slight gradual decrease of the C/N ratio was shown for the MUR-U2 profile (Figure 3C). In general, the C/N ratio in Technosols was lower than in Podzols throughout the profile, which is likely a result of additional N input, as it is often reported for urban soils in comparison to the natural references [45,46].

The content of heavy metals in urban soils was higher than in the natural Podzol (*p* = 0.01088). Topsoil contents of Pb, Zn, and Ni in urban soil were also above the health thresholds (maximum permissible concentration) [47,48]; however, the values were much lower than reported for the industrial areas in the region [8,11]. Phosphorus concentrations in urban topsoils were 2–3 times higher than in the natural areas, which was especially evident for the MUR-U1 profile at the central part of the city. Likely, a long-term residential activity contributed to the additional input of phosphorous, which is also often reported for urban soils and sometimes even referred to as P-pollution of urban soils [49,50]. Potassium contents in urban soils were also 10–20% higher than in Podzol. Finally, the bulk density of urban topsoil was higher than in the natural soil, whereas subsoil bulk density was comparable in all three profiles (Table 2).

Urbanization resulted in substantial and complex changes in soil properties. Additional input of carbon and nutrients as well as shifting soil pH increased soil fertility and likely created favorable conditions for microbial growth. At the same time, a negative effect of over-compaction and increased content of heavy metals on soil microbial biomass, activity, and diversity could be expected. Qualitative and quantitative microbiological analyses revealed the consequences of these urbanization outcomes on the soil microbiome.

### 3.3. Microbiological Properties

#### 3.3.1. Number of Gene Copies

##### Archaea

**Figure 4:**
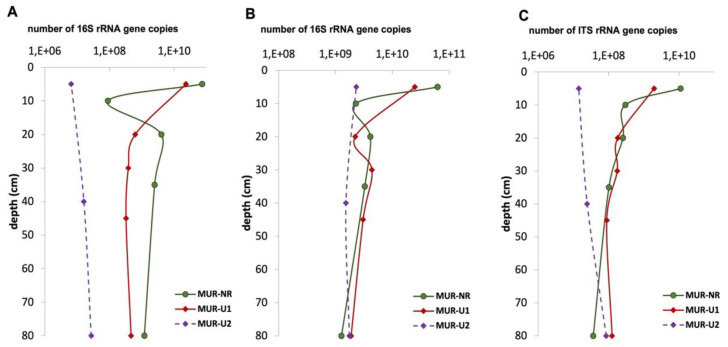
The number of 16S rRNA genes copies of archaea (**A**), bacteria (**B**), and ITS rRNA genes copies of fungi (**C**).

The number of 16S rRNA gene copies of archaea in the O horizon of the natural Podzol was 7.28 × 10^10^, which was three times higher than in the Au of the urban MUR-U1 profile. In the E horizon of the MUR-NR profile, the number of gene copies decreased almost three orders of magnitude compared to O horizon, but it further increased to 10^6^–10^7^ gene copies/g soil in the mineral subsoil horizons. In the MUR-U1 profile, the number of 16S rRNA genes copies of archaea gradually decreased down the profiles with the most significant difference between Au and BC horizons. Surprisingly, the content of archaea in the buried Aub horizon of the urban soil MUR-U1 was similar to the underlining mineral horizons and was considerably (60 times) less than in the surface Au horizon, even though their chemical properties were comparable (see Figure 3). The lowest content of archaea (on average, about 10^8^ gene copies/g soil throughout the profile) was obtained for the suburb urban site MUR-U2, where a slight increase was shown for the subsoil ABub horizon in comparison to the topsoil (Figure 4A).

##### Bacteria

The profile distribution of the number of 16S rRNA bacterial gene copies in natural Podzol was similar to the archaea distribution with the rapid decrease from O (6.25 × 10^10^) to E (2.28 × 10^9^) horizons followed by the second maximum in illuvial Bhs horizon (4.13 × 10^9^) and further gradual decrease in the mineral subsoil horizons. A similar ‘bimodal’ profile distribution was shown for the MUR-U1 urban soil, where both maximums referred to the surface and buried organic Au horizons. In MUR-U2 soil, the number of bacteria genes copies in Au and ABub horizons was higher than in the technogenic BCu; however, the difference between the two horizon was rather small. Overall, urban soils contained fewer bacteria gene copies in the topsoil compared to the natural soils; however, in the subsoil horizons (on average below 30 cm), the content of bacteria in urban soils was similar or even higher than in the natural reference (Figure 4B).

##### Fungi

The number of ITS rRNA gene copies of fungi in natural MUR-NR topsoil (1.11 × 10^10^) was six times and five orders of magnitude higher than in urban MUR-U1 and MUR-U2, respectively; however, the opposite was shown for the subsoil layers deeper than 50 cm, where the number of fungi gene copies in urban soils was two-three times higher than in the natural reference (Figure 4C). The values obtained for the middle part of the profile (between 10 and 50 cm) were rather close for all three profiles, with the minimum number for MUR-U2 and negligible differences between MUR-U1 and MUR-NR soils.

#### 3.3.2. Microbial Biomass

**Figure 5:**
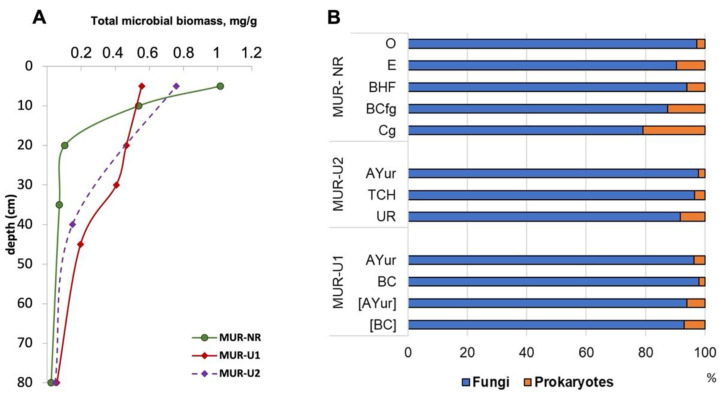
Profile distribution of the total microbial biomass (**A**) and fungi/ prokaryotes portion (**B**).

The highest total microbial biomass was obtained for the natural MUR-NR topsoil, where it was up to two times higher than in the urban soils. However, in the natural soils, microbial biomass decreased exponentially down the profile (the difference between 5 and 20 cm depths was one order of magnitude), whereas in the urban soils, the decline in microbial biomass down the profile was more gradual. As a result, urban subsoils had higher microbial biomass compared to the natural reference (Figure 5A). In all profiles, microbial biomass was dominated by fungi, whose portion ranged from 79 to 98%. The highest contribution (96–98%) was obtained for the topsoil horizons, whereas in the mineral subsoil horizons, the portion of fungal biomass decreased. The difference between topsoil and subsoil was more noticeable for the natural soil, whereas in the urban soils, the numbers were rather close, especially for the MUR-U1, where the difference between surface Au and buried Aub was only 2% (Figure 5B). 

#### 3.3.3. Fungal Biomass

The proportion of mycelium, an active component of fungal biomass, varied from 28 to 76% in urban soils and from 0 to 80% in the natural soil. The minimum of mycelium (up to complete absence) was found in the subsoil horizons, whereas the topsoil horizons were rich in fungal hyphae. The length of fungal mycelium decreased down the profile, generally following the patterns obtained for total microbial biomass in urban and natural soils. The average length of the fungal mycelium in Urbic Techosols soils was half from the results obtained for the natural Albic Podzol (*p* = 0.01079). The proportion of thin (less than 3 μm in diameter) mycelium was also two times higher in the urban area—43 and 26% for the urban and natural soils, respectively. The number of unicellular fungal propagules (spores and yeasts) in the investigated soils was 104–105 cells/g soil. The forms of small size (2–3 microns) were dominating, and their contribution increased from the topsoil (68–93% in the urban soil and 88% in the natural soil) to subsoil horizons (up to 100%). Large propagules with a diameter of 5–7 µm were rare (less 103 cells/g), and they were found exclusively in urban topsoil horizons. In the mineral layers, fungi were almost entirely represented by unicellular propagules (spores and yeasts). About 81% of the propagules had a round shape with a smooth surface, 4% were round and rough, 11% were oval with a smooth surface, and 4% were oval with irregularities (Table 3).

#### 3.3.4. Prokaryote’s Biomass

The maximum biomass of prokaryotes was obtained for the natural topsoil—24.83 µg/g soil in the O horizon, which was 20% higher than in urban topsoils (*p* = 0.006993) (Table 4). A gradual decrease down the profile was shown for the biomass of prokaryotes in MUR-NR and MUR-U2 sites, whereas the profile distribution in MUR-U1 had the second maximum corresponding to the buried [Au] horizon. The biomass of prokaryotes was mainly represented by unicellular forms; however, in some samples, the actinomycete mycelium reached 9–19%. The length of the mycelium of actinomycetes in the different soil horizons ranged from 1.04 to 75.06 m/g in the urban soil and from 2.42 to 83.82 m/g in the natural soil (*p* = 0.006851). For all mineral horizons (except for the E profile MUR-NR), mycelial prokaryotes had hyphae no longer than 11.45 m/g of soil, while in the topsoil horizons, more than 40.14 m/g of soil. Most (up to 55%) of prokaryotic cells were small nanoforms.

## 4. Discussion

### 4.1. The Effect of Urbanization on Soil Chemical and Microbial Properties in the Arctic

The principal changes in the soils of Murmansk induced by urbanization included shifting pH to neutral and an increase in carbon and nutrients’ contents. This is in agreement with the previous studies, which used remote sensing and spatial modeling to show a positive effect of urbanization on the topsoil C stocks with the highest C stocks in the recreational and residential zones [31]. Neutralization of the naturally acidic soils is a typical urbanization effect previously described for many cities in polar and boreal zones [51,52,53]. Dust deposition from building construction, as well as the implementation of the de-icing reagents, are the main sources of the additional calcium input, shifting soil pH [54,55]. In both investigated urban sites, pH_H2O_ was significantly higher than in the natural soil, with a more remarkable shift for the MUR-U2 site, which was recently developed and exposed to more intensive management. Topsoil SOC content in urban soils was comparable to the previous studies for Murmansk [11,31], Salekhard [56], and some other polar cities [46,57] and higher than reported for the natural Podzols in the region [29,36]. In the investigated MUR-NR site, however, SOC content in the topsoil O horizon was much higher than in the urban sites due to slow decomposition of the plant residuals in Subarctic conditions resulting in peat formation. An additional N input identified by a lower C/N ratio is also a typical feature for urban soils [45,58]. High P contents in urban areas are usually explained by fertilization, stormwater or feces of domestic animals [59,60], but for the Murmansk soils, the effect of the mining activities can also be considerable [61]. The combined effect of C, N, and P inputs increases the stoichiometric C/N/P ratio with a considerable effect on soil microbiome [58,62] which is supported by high correlation values r = 0.68–0.95. In the urban soils of Murmansk, a decrease in the number of gene copies of all groups of microorganisms was noted in comparison with the natural soil. The fungal biomass in the urban soils was less than in the natural Podzol soils of the Kola Peninsula [63,64] but 2–4 times higher compared to the other settlement of the region [65]. This is likely explained by the abundance and availability of organic matter [66] and relatively low pollution by heavy metals compared to Monchegorsk or Apatity settlements [67]. The previous studies also did not report considerable pollution in the residential areas of Murmansk, whereas a higher content of heavy metals was shown for the industrial areas and traffics zones in comparison to the natural sites [11,30]. As a big city, Murmansk is exposed to a stronger urban heat-island effect than any other settlement in the region, which in combination with the warming effect of the Gulf Stream creates favorable climatic conditions for soil microbiota [68]. For example, the length of the fungal mycelium in the studied soils was almost 1.5 times higher compared to Novaya Zemlya, a more northern location [69]. The proportion of thin (less than 3 μm in diameter) mycelium of the studied soils is relatively small compared to the urban soils of Apatity and settlements on the Barents Sea coast of the Kola Peninsula, where the proportion of fine mycelium exceeded 40% [65,70]. The number and biomass of prokaryotes in the studied soils of Murmansk were also 1.5–2 times higher than in other Arctic territories—Taimyr [71], Novaya Zemlya [69], and Franz Josef Land [72,73]. The length of actinomycete mycelium reached tens of meters, which is comparable to Apatity [65] and confirms the assumption that the proportion of this order of Gram-positive bacteria is relatively high for polar ecosystems [74,75]. Up to 55% of the prokaryotic cells in the studied soils of Murmansk were represented by small nanoforms, which is also typical for polar ecosystems [76,77]. Thus, the soil microbiota of Murmansk possessed features typical for a polar environment, as well as specific features induced by the urbanization effect.

### 4.2. Subsoil Contribution to Microbiota in Urban and Natural Soils in Subarctic

The difference between urban and natural soils was even more remarkable when the profile distributions of soil chemical and microbial properties were compared. At the natural soil, a classical for Podzols ‘bimodal’ distribution with the maximums in organic and illuvial horizons was obtained for SOC, nutrients and some heavy metals. This distribution is explained by the slow accumulation of organic matter and vertical migration of organic matter, iron and silt particles being the dominating soil-forming process in cold, humid conditions. The distribution of different groups of microorganisms (archaea, bacteria, and fungi) generally followed this pattern with the local reduction in the number of microorganisms gene copies from the surface to deep horizons [78,79]. The distribution of the fungal biomass in the natural Podzol generally followed the bimodal distribution of SOC with the highest number of the ITS rRNA gene copies of fungi and the highest fungi/prokaryotes ratio observed in the O horizon. This may be due to the mycorrhizal mycobiota, which have the greatest extent of mycelium in the surface layers [80]. The greatest length of mycelium and number of large propagules (5–7 μm in diameter) were also shown for the O horizon, where the abundance of organic matter ensures the greatest taxonomic diversity of mycobiota, which is supported by high correlation values r = 0.68–0.72 [81]. The deeper layers were depleted in fungal propagules due to the lack of organic matter, limited access to the atmospheric oxygen, and the low number of fine roots required for symbiosis [82,83]. The total biomass decreased exponentially down the profile and almost 60% of the total biomass was concentrated in top 5–10 cm.

In the urban areas, profile distribution was mainly driven by the land-use history and land management. The pH_H2O_ was slightly acidic to neutral throughout the profile reaching slightly alkaline values in the technogenic BCu horizon of the MUR-U2 site. The second maximum of SOC content was considerably deeper than in the natural Podzol and referred to the buried Aub in MUR-U1 or to the ABub horizon in MUR-U2. The contribution of urban subsoils, including buried horizons and cultural layers, which is often ignored by soil surveys, can be very considerable in the areas with a long residential history. For example, subsoil SOC stocks in Moscow [84], Krakow [85], and Veliky Novgorod [86] were up to one order of magnitude higher compared to the topsoils. Cold conditions of Murmansk hamper the mineralization of organic matter and can contribute to its accumulation in urban soils for a long period of time. Profile distribution of N also had the second maximums in Aub and ABub, which confirms its anthropogenic origin (e.g., from fossil fuel combustion, sewage water or wastes’ deposition) [45,87]. At the same time, heavy metals were mainly accumulated in the surface layers, which indicate air deposition from the traffic zones, industrial and mining activities that are the main pollution sources in the region [8,10]. As a result, urban subsoils with neutral pH, high content of organic matter and nutrients, and low pollution levels provided favorable conditions for microbial development. Although the total microbial biomass in urban soils decreased down the profile, the pattern was more gradual compared to the natural soils—only one-third of the total biomass was concentrated in the top 5–10 cm. The number of the rRNA gene copies of fungal and bacterial in the subsoils layers deeper than 30 cm was higher in the urban soil (mainly in MUR-U1) than in the natural soil horizons at the same depth. The number of fungi and bacteria gene copies in the horizons was positively correlated with the SOC content, which is in good coherence with the previous studies [73,78,88]. The fungi/bacteria ratio in urban subsoils was almost two times higher than in natural soils and never decreased below 90%. Therefore, urban subsoils can be considered an important niche for soil microbiomes development in Arctic and Subarctic conditions.

## 5. Conclusions

Urbanization in the Subarctic and Arctic is becoming a ‘hot’ topic due to complex and still poorly known consequences for vulnerable ecosystems. A comparative analysis of the soil microbiome in natural Podzol and urban soils of Murmansk showed that the effect of urbanization is not limited to pollution and physical disturbance. Moreover, the effect of urbanization on topsoil and subsoil microbial properties was different. In urban topsoils, microbial biomass and the number of rRNA genes’ copies of bacteria and fungi were less compared to the natural soils. However, the opposite was shown for the subsoil, where neutral reaction, high carbon and nutrients content, and low pollution created favorable conditions for microbial development.

Total biomass, the number of fungi and bacteria gene copies, and fungi/bacteria ratio in urban subsoils were higher than in the natural soils at the same depth. We demonstrated that urbanization in Arctics increased the activity and abundance of soil microorganisms. This outcome shall not be directly interpreted as a positive effect of urbanization on soil microbiome since the taxonomic and functional composition of microbial communities in urban soils can be very diverse. At the same time, urban subsoil was shown as a potential ecological niche for soil microbiome development and a source of important ecosystem services, such as nutrient cycling and biodiversity control. The importance of the ecosystem services and disservices provided by the urban microbiome will further increase following the global warming scenarios and shall be considered for sustainable soil management in Arctic cities.

## Figures and Tables

**Table 1:** Information about primers and standards for qPCR.

Target Group of Process	Target Gene	Primer Name	Primer Sequence (F, R)	Standard Source	Reference
Total Bacteria	16s rRNA	Eub338 Eub518	ACCTCTACGGGAGGCAGCAG ATTACCGCGGCTGCTGCTGG	Escherichia coli	Fierer et al., 2005
Total Archea	16s rRNA	915f 1059r	AGGAA TTGGC GGGGG AGCAC GCCAT GCACC WCCTC T	Strain FG08 Halobacterium salinarum	Yu et al., 2005
Total Fungi	ITS region	ITS1f 5.8 s	TCC GTA GGT GAA CCT GCG G CGC TGC GTT CTT CAT CG	Saccharomyces cerevisiae Meyen 1B-D1606	Fierer et al., 2005

**Table 2:** Chemical and physical properties of urban and natural soils in Murmansk area.

Horizon	BD (g cm^−3)^	P (mg kg^−1^)	K (mg kg^−1^)	Heavy Metals (mg kg^−1^)					
				Pb	Zn	Co	Cd	Cu	Ni
MUR-NR									
O	0.16	348	75	34.5	50.9	3.3	0.12	21.9	29.8
E	1.45	64	64	24.5	13.8	2.3	0.3	1.1	7.4
Bhs	1.2	554	65	29.2	48.2	9.1	0.25	9.4	22.5
BCs	1.22	260	57	31.5	60.3	14.9	0.55	11.2	39.5
Cg	1.62	675	44	22.7	59.6	15.8	0.31	27.9	44.3
MUR-U1									
Au	0.9	1054	84	66	149.8	13.5	0.39	20.3	38.4
BC	1.38	1002	64	21.7	58.1	11.8	0.35	6.1	26.3
Aub	1.35	401	55	32.4	94.2	11.4	0.37	30.4	25.5
BC	1.34	428	105	24.4	102.1	10.8	0.42	8.2	29.6
C	1.6	550	50	20	60	11	0.3	9	28
MUR-U2									
Au	0.85	669	121	35.8	136.2	15.2	0.3	22.7	44.7
BCu	1.41	340	102	22.3	65.7	13.5	0.17	16.1	37.8
ABub	1.5	610	110	25	80	15	0.25	22	45

**Table 3:** Fungal biomass and structure in urban and forest-tundra soils.

Horizon (Depth, cm)	Total Biomass of Fungi, µg/g	Biomass of Mycelium, µg/g (d = 3 µ)	Biomass of Spores, µg/g	Share of Mycelium in the Total Biomass, %	Number of Spores (Diameter, µ)			
					2, Unit/g × 10^4^	3, Unit/g × 10^4^	5, Unit/g × 10^3^	7, Unit/g × 10^3^
MUR-U1								
Au (0–10)	0.535 ± 0.096	0.324 ± 0.038	0.211 ± 0.038	60.6	27.62 ± 3.79	9.03 ± 1.24	1.39 ± 0.24	-
BC (10–35)	0.456 ± 0.082	0.347 ± 0.041	0.109 ± 0.020	76.1	13.38 ± 1.84	5.49 ± 0.74	-	-
Aub (35–45)	0.391 ± 0.070	0.213 ± 0.025	0.178 ± 0.032	54.5	10.36 ± 1.42	12.26 ± 1.70	-	-
BC (45–55)	0.189 ± 0.034	0.100 ± 0.012	0.089 ± 0.016	52.9	13.38 ± 1.84	3.87 ± 0.51	-	-
C (55–90)	0.053 ± 0.009	0.015 ± 0.002	0.038 ± 0.007	28.3	6.905 ± 0.948	1.29 ± 0.14	-	-
MUR-U2								
Au (0–10)	0.741 ± 0.133	0.418 ± 0.049	0.323 ± 0.058	56.4	11.22 ± 1.54	16.13 ± 2.24	2.78 ± 0.47	0.693 ± 0.124
BCu (10–55)	0.141 ± 0.025	0.068 ± 0.008	0.073 ± 0.013	48.2	8.632 ± 1.18	3.87 ± 0.54	-	-
ABub (55–90)	0.044 ± 0.008	0.022 ± 0.003	0.022 ± 0.004	50.0	4.316 ± 0.592	0.645 ± 0.090	-	-
MUR-NR								
O (0–7)	0.992 ± 0.179	0.733 ± 0.086	0.259 ± 0.047	73.9	12.52 ± 1.77	16.42 ± 2.28	3.13 ± 0.54	1.04 ± 0.190
E (7–20)	0.522 ± 0.094	0.344 ± 0.040	0.178 ± 0.032	65.9	17.26 ± 2.44	9.67 ± 1.34	1.04 ± 0.18	-
Bhs (20–30)	0.094 ± 0.017	0.025 ± 0.003	0.069 ± 0.012	26.6	9.500 ± 1.34	3.22 ± 0.45	-	-
BCs (30–55)	0.063 ± 0.011	0.029 ± 0.003	0.034 ± 0.006	46.0	0.333 ± 0.047	2.90 ± 0.40	-	-
Cg (55–90)	0.019 ± 0.003	-	0.019 ± 0.003	0.0	0.194 ± 0.027	1.61 ± 0.22	-	-

**Table 4:** Prokaryote’s biomass.

Horizon (Depth, cm)	Number, Unit/g, ×10^8^	Biomass of Oligocellular Prokaryotes, µg/g of Soil	Length of Actinomycetes Mycelium, m/g	Biomass of Actinomycetes Mycelium, µg/g	Portion of Mycelium in the Total Biomass, %	Total Prokaryotes Biomass, µg/g of Soil	Average Prokaryotes Biomass in the Soil Profile, µg/g of Soil
MUR-U1							
Au (0–10)	8.38 ± 1.23	17.66 ± 2.66	71.46 ± 10.8	2.67 ± 0.45	13.1	20.33 ± 3.66	11.03 ± 1.99
BC (10–35)	4.21 ± 0.62	8.87 ± 1.33	1.04 ± 0.16	0.038 ± 0.0064	0.4	8.90 ± 1.60	
Aub (35–45)	6.33 ± 0.93	13.33 ± 2.00	40.14 ± 6.07	1.50 ± 0.25	10.1	14.83 ± 2.67	
BC (45–55)	3.27 ± 0.48	6.88 ± 1.04	7.76 ± 1.17	0.29 ± 0.05	4.0	7.17 ± 1.29	
C (55–90)	1.86 ± 0.27	3.94 ± 0.59	-	-	0.0	3.94 ± 0.59	
MUR-U2							
Au (0–10)	6.80 ± 1.00	14.32 ± 2.16	75.06 ± 11.35	2.55 ± 0.43	15.1	16.87 ± 3.04	9.64 ± 1.74
BCu (10–55)	3.82 ± 0.56	8.05 ± 1.21	4.71 ± 0.71	0.16 ± 0.03	2.0	8.21 ± 1.48	
ABub (55–90)	1.86 ± 027	3.85 ± 0.58	-	-	0.0	3.85 ± 0.58	
MUR-NR							
O (0–7)	10.91 ± 1.60	22.97 ± 3.45	54.82 ± 8.29	1.86 ± 0.31	7.49	24.83 ± 4.47	13.51 ± 2.43
E (7–20)	5.79 ± 0.85	12.20 ± 1.84	83.82 ± 12.68	2.84 ± 0.48	18.9	15.04 ± 2.71	
Bhs (20–30)	5.05 ± 0.74	10.65 ± 1.60	4.07 ± 0.62	0.14 ± 0.02	1.3	10.79 ± 1.94	
BCs (30–55)	4.13 ± 1.84	8.70 ± 1.31	11.45 ± 1.73	0.39 ± 0.07	4.29	9.09 ± 1.64	
Cg (55–90)	2.46 ± 1.09	5.20 ± 0.78	2.42 ± 0.37	0.08 ± 0.13	1.5	5.28 ± 0.95	

## Data Availability

No new data were created or analyzed in this study. Data sharing is not applicable to this article.
